# Intelligent Bio-Responsive Fluorescent Au–shRNA Complexes for Regulated Autophagy and Effective Cancer Bioimaging and Therapeutics

**DOI:** 10.3390/bios11110425

**Published:** 2021-10-28

**Authors:** Weijuan Cai, Liang Yin, Hui Jiang, Yossi Weizmann, Xuemei Wang

**Affiliations:** 1 State Key Laboratory of Bioelectronics (Chien-Shiung Wu Lab), School of Biological Science and Medical Engineering, Southeast University, Nanjing 210096, China; 230188127@seu.edu.cn (W.C.); sungi@seu.edu.cn (H.J.); 2 Department of Endocrinology and Metabolism, Shunde Hospital of Southern Medical University, Shunde 528300, China; yinliang151@sina.com; 3 Department of Chemistry, Ben-Gurion University of the Negev, Beer-Sheva 8410501, Israel

**Keywords:** bio-responsive fluorescent complexes, shRNA delivery, LncRNA MALAT1, cancer cells bioimaging, therapeutics, autophagy

## Abstract

The long non-coding RNA (lncRNA) MALAT1 acts as an oncogene. RNA interference (RNAi) is an effective method to control the expression of specific genes and can be used for the treatment of tumors, but an effective and safe carrier system is a significant obstacle to gene therapy. Herein, we explored the possibility of constructing an in situ bio-responsive self-assembled fluorescent gold-short hairpin RNA nanocomplex (Au–shRNA NCs) delivery system by co-incubating gold and MALAT1-shRNA for precise hepatocellular carcinoma (HCC) imaging and treatment. Due to the characteristics of the cancer microenvironment, Au–shRNA NCs self-assembled in HCC cells (HepG2) but did not occur in control cells (L02) under the same conditions. The in situ bio-responsive self-assembled Au–shRNA NCs delivery system can realize cancer cell bioimaging and promote cell uptake and endosomal escape mechanism, thereby realizing effective transfection. They effectively silenced target gene MALAT1, and with the downregulation of MALAT1, we found that several molecules involved in autophagic flux were also regulated. In vitro and tumor-bearing mouse model experiments demonstrated that the as-prepared fluorescent Au–shRNA NCs can readily realize tumor bioimaging and effectively silence the target gene MALAT1, and those autophagy-related pathway molecules were significantly downregulated, thereby exerting a tumor suppressor efficiency. This raises the possibility of realizing accurate multi-scale bio-imaging from the molecular-level with targeted gene-recognition to cancer cell imaging as well as in vivo tumor tissue imaging for the simultaneous precise cancer therapy.

## 1. Introduction

Long noncoding RNAs (lncRNAs) are more than 200 nucleotides in length [1,2] and participate in numerous physiological and pathophysiological activities such as carcinogenesis and autophagy [3,4,5]. Aberrant expression or dysfunction of lncRNAs is closely associated with various diseases [6,7,8,9]. Recently, research findings have illustrated that lncRNAs may also be involved in remodeling the tumor microenvironment and in tumor metastasis [10]. Metastasis-associated lung adenocarcinoma transcript 1 (MALAT1) is one of the star molecules of lncRNA, which has been determined to participate in various processes including cell apoptosis and proliferation [11]. As reported in several studies, MALAT1 serves as a potentially valuable biomarker in cancer diagnosis and prognosis [12]. Meta-analyses have shown the association between high MALAT1 level and poor clinical outcomes [13,14]. In addition, it is reported that MALAT1 is a mutation factor associated with the occurrence of hepatocellular carcinoma (HCC) [15]. However, how MALAT1 can be used to target HCC therapeutically and the underlying mechanism remain largely unknown.

RNA interference (RNAi) is considered to be a gene silencing phenomenon present in most eukaryotic cells. RNAi has the potential to treat almost any disease by using appropriate sequences to silence the expression of virtually any target gene [16]. Small interfering RNAs (siRNAs) are effector molecules in the process of RNAi [17]. Although the simplest RNAi method is cytoplasmic delivery via siRNA oligonucleotides, the technology is restricted to cells suitable for transfection and is mainly used in transient expression study. An exogenously introduced expressing short hairpin RNA (shRNA) has similar functions to siRNA and can also exert RNAi effects [18]. The shRNA can be converted into siRNA in the cell to exert a gene silencing effect and achieve long-term knockdown of the targeted gene. In addition, shRNA is being rapidly developed into a new avenue for gene function analysis and a new treatment modality. However, this approach faces significant challenges in achieving tissue specificity and the safe and effective delivery of shRNA.

Several obstacles related to systemic shRNA delivery include clearance by the reticuloendothelial system, the complex extracellular matrix and environment around tumor cells, off-target effects, and poor cellular uptake [19,20]. Given the above, in this study, we designed in situ bio-self-assembled Au–shRNA nanocomplexes (Au–shRNA NCs), then examined their ability to silence target gene MALAT1 and their effectiveness in tumor bioimaging and treatment. Currently, the alarming incidence of chronic hepatitis B virus and C has led to most HCCs, and these cases have become the third leading cause of cancer death [21,22]. HCC has been mechanistically explored in some studies, but these efforts have not improved survival. Thus, it is vital to exploit the molecular mechanisms that regulate the metastatic behavior of HCC to develop new therapies that target HCC. More importantly, this approach can also accurately help real-time tumor monitoring and bioimaging. MALAT1 has unique mechanisms of action in different types of cancer [23], which acts as an oncogenic lncRNA in HCC and is often highly expressed [24,25]. Autophagy is a conservative lysosome-mediated intracellular catabolic process, which is very important for cellular homeostasis [26]. Studies have shown that lncRNA plays a crucial role in the process of autophagy [27]. MALAT1 promotes proliferation and metastasis of invasive pancreatic cancer through autophagy stimulation [28]. Silencing MALAT1 can inhibit chemically induced autophagy, while overexpression of MALAT1 can promote autophagy in gastric cancer [11]. However, whether silencing MALAT1 affects HCC cell autophagy is unclear.

The bioimaging process is the most direct and effective way for biological structure and function research. It uses optical or electron microscopes to directly obtain microstructure images of biological cells and/or tissues, and understands various physiological processes of biological cells through the analysis of the resulting images [29]. Furthermore, applying new materials such as nanomaterials makes imaging technology play a more significant role [30]. At present, the growing trend of bioimaging technology also requires the advancing direction of molecular imaging technology not only for clinical diagnosis and treatment, but also for new drug development and basic research of human science.

Herein, we explored a new approach of the systemic shRNA delivery for lncRNA MALAT1-regulated autophagy via the in situ synthesis of bio-self-assembled Au–shRNA NCs in HCC cells/or in vivo tissues. Biological imaging techniques such as confocal, transmission electron microscopy (TEM), and atomic force microscopy (AFM) help us observe that the ability of the as-prepared fluorescent Au–shRNA NCs to regulate target gene MALAT1 on autophagy and silence MALAT1, and demonstrate its efficiency for the real-time imaging and monitoring of tumor treatments. This raises the possibility of the in vivo utilization of this novel Au–shRNA NC delivery system via RNAi to inhibit HCC progression and realize effective HCC imaging and therapy.

## 2. Materials and Methods

### 2.1. Cell Culture

In the research, we purchased human hepatocarcinoma cell lines (HepG2, SMMC-7721) and control cells (human embryonic liver L02) from ATCC (Manassas, VA, USA). L02, HepG2, and SMMC-7721 cells were cultured with DMEM (4.5 g/L glucose) supplemented with 1% penicillin/streptomycin and 10% fetal bovine serum (all from Gibco, Australia). The culture conditions were strictly at 37 °C, 5% CO_2_, and a 95% humidity environment.

### 2.2. Patients and Specimens

This study was approved by the First Affiliated Hospital, Shihezi University School of Medicine. From May 2019 to January 2021, a total of 30 tumor tissues and matched normal adjacent tissues were collected from HCC patients registered to our hospital through surgical resection. Importantly, all tissues were snap-frozen in liquid nitrogen until further use. We excluded patients from receiving chemotherapy or radiotherapy preoperatively or postoperatively. All human samples were obtained with the patients’ written consent. In Table S1, the clinicopathological characteristics of the HCC patients are listed in detail.

### 2.3. qRT-PCR

We used TRIzol reagent (Invitrogen, USA) to isolate the total RNA from frozen tissue. Before further experiments, the purity and concentration of the extracted RNA samples were quantified using NanoDrop ND-1000 equipment. The steps described in the Hairpin-it qRT-PCR Kit (GenePharma Co., Shanghai, China) were followed to reverse-transcribe the total RNA (2 μg) of each sample. Then, the qRT-PCR ran according to the qRT-PCR Kit instructions, and the CT values were obtained after the end of the reaction. Finally, the relative changes in gene expression were calculated by the 2^−ΔΔCT^ method. In this experiment, GAPDH was used as an internal control. The primer sequences were purchased from Invitrogen (Waltham, MA, USA) and are shown in Table S2. All PCR runs were performed in triplicate.

### 2.4. MTT Cytotoxicity Assessment and Cytostatic Test

Initially, deionized water was used to dilute the HAuCl_4_ (Shanghai Yuanye Bio, China, CAS:27988-77-8, pH = 7.2) to create solutions with the appropriate concentrations for the toxicity tests. Briefly, we took the prepared liquid with a concentration of 10 nM HAuCl_4_, and then diluted it according to the experimental design. The final concentration gradient was 0, 0.5, 1, 5, 10, 30, 50, 100, 200, and 500 μM for the experiment. Then, we used trypsin to digest L02 and HepG2 cells, and 200 μL complete medium containing approximately 4000 cells was placed in 96-well plates. Following this, the experimental arrangement was incubated with different concentrations of HAuCl_4_ for 48 h and the experiment proceeded following instructions in the MTT Kit where the absorbance measurement needs to be performed at a wavelength of 490 nm. Next, according to the concentration range provided in the instructions, the best shRNA concentration and silencing effect were determined. Finally, HepG2 cells in the logarithmic growth phase were seeded in a single cell suspension in a 96-well plate, and a cytostatic test was carried out for five days. After 24 h of incubation, complete fresh medium was added to the cells, followed by the addition of HAuCl_4_ and shRNA1 successively, and then co-incubated for 0, 1, 2, 3, 4, and 5 days. The concentrations of shRNA1 and HAuCl_4_ were 3 ng/µL and 5 μM, respectively. At each time interval point, we analyzed the absorbance value and drew the cell growth curve. Each experiment needed to ensure that three biological replicates were used.

### 2.5. Wound Healing Assay

A 6-well plate to culture cells was used (the number of cells per well is the same), and when the cell density reached 80–90%, a p200 pipette tip was employed to scrape the cells. Different groups of cells at 0, 12, 24, and 48 h were processed, and their images were captured at the same time interval, respectively. Finally, all images were analyzed using ImageJ software. In this experiment, the concentrations of shRNA1 and HAuCl_4_ were 3 ng/µL and 5 μM in the Au–shRNA1 NC group, respectively.

### 2.6. In Situ Biosynthetic Au–shRNA1 NCs

Adherent HepG2 cells in culture were exposed to HAuCl_4_ solution at a final concentration of 5 μM. The cells were gently shaken to mix the solution well with the medium. Then, the cells were put back into the cell incubator. After a few minutes, the cells were settled, and the shRNA1 plasmid (ViGene Biosciences, China) that silences MALAT1 was added to the medium for co-incubation. After incubating for at least 24 h, the medium was first discarded. Next, the cells were washed three times with PBS and trypsinized for 1–2 min. The remaining trypsin solution was removed, and 2 mL PBS was added, and the sample was centrifuged in a sterile centrifuge at 1500 rpm for 3 min. After that, the supernatant was removed, and deionized water was added to resuspend the cells. As previously described [31], the repeated freeze–thaw method was used to prepare the cell extracts for further characterization.

### 2.7. Cellular Uptake and Colocalization Studies

HepG2 cells were plated (1 × 10^6^ cells per well) on a laser confocal culture dish, and the cells were first pretreated with various endocytosis inhibitors for about 1 h, then, gold salt and shRNA were added sequentially and incubated with the cells for 6 h. The concentrations of the inhibitors was as follows: 37 mg/mL methyl-β-cyclodextrin, 10 μg/mL chlorpromazine, 10 mg/mL rottlerin, 200 μg/mL genistein, and 5 μg/mL filipin III (Sigma-Aldrich, MO, USA). After 6 h of co-incubation, the HepG2 cells were washed three times with PBS and fixed with 4% paraformaldehyde for 30 min. The cell nucleus was stained with DAPI (Beyotime, Shanghai, China). Finally, a confocal microscope was used to image the sample with 488 nm (Leica, Wetzlar, Germany).

To observe the subcellular localization of Au–shRNA NCs, gold salt and the shRNA were co-incubated with HepG2 cells at 37 °C for 12 h. The specific experimental process was the same as that above-mentioned. Endosomes and lysosomes were labeled by Lysotracker Red for 30 min and washed with PBS (three times), followed by nuclei staining with DAPI for 3 min. The images were obtained by confocal microscopy (Leica, Wetzlar, Germany).

### 2.8. Transmission Electron Microscopy (TEM)

We first diluted the Au–shRNA1 NC extracts with deionized water, then dropped it on the copper grid and waited for it to dry naturally. We used TEM (JEM-2100, JEOL Ltd., Tokyo, Japan) to characterize the size and distribution confirmation of the in situ formation of Au–shRNA1 NCs. In addition, we also evaluated the structure of lysosomes and autophagosomes and/or autolysosomes through bio-TEM. Briefly, HepG2 cells were seeded in 6-well plates and processed according to different groups. After 24 h of incubation, the cells were collected, the culture medium was discarded, and the electron microscope fixation solution (glutaraldehyde) was added for fixation. Finally, the cells were observed under TEM (Hitachi-HT7700, Hitachi High-Tech Corporation, Tokyo, Japan) and collected for image analysis.

### 2.9. Atomic Force Microscopy (AFM)

Before adding the sample, the mica flakes (15 mm × 15 mm) were immersed in Mg^2+^ solution (10 nM MgCl_2_ solution) for 5 min in advance. Next, the lysis sample (10 µL) with deionized water was deposited onto freshly cleaved mica to adsorb for 5 min, rinsed gently with distilled water, and then we waited for a few minutes until the specimen was dry. The morphology and characteristics of Au–shRNA1 NCs were characterized by AFM (Bruker Dimension Icon, Bruker, Billerica, MA, USA). The concentrations of shRNA1 and HAuCl_4_ added to the original extraction solution were 3 ng/µL and 5 μM, respectively.

### 2.10. Fluorescence Confocal Microscopy

HepG2 cells were seeded on a laser confocal culture dish, processed according to different groups, and incubated for 24–48 h. Next, HepG2 cells were fixed (4% paraformaldehyde) for 30 min, washed with PBS three times, and then permeabilized with 0.3% Triton X-100 for 20 min. Finally, DAPI was added to stain the HepG2 cell nuclei (blue). The images were obtained by confocal microscopy (Leica, Wetzlar, Germany).

### 2.11. Western Blot Analysis

Western blotting was performed as described previously [31,32]. Briefly, whole-cell lysates containing approximately 40 μg of protein were loaded on 10% sodium dodecyl sulfate-polyacrylamide gel electrophoresis. Then, the transfer of the PVDF membrane was carried out by the electrotransfer method. After that, the membranes were incubated with the antibodies listed in Table S3. According to the experiment, we added the appropriate secondary antibody and incubated it together, then captured the blots on the Bio-Rad chemiluminescence imager. We then used ImageJ software for relative protein content analysis. The experimental internal reference was GAPDH and repeated three times.

### 2.12. GFP-(Microtubule-Associated Protein 1 Light Chain 3 (LC3)/Lysosomal-Associated Membrane Protein2 (LAMP2)/p62 Staining

Different groups of cells were processed according to the experimental conditions and cultured on laser confocal Petri dishes. After incubation with shRNA1 and gold salt for 24–48 h, HepG2 cells were washed twice with cold 1 × PBS, then placed in the fixative solution (4% paraformaldehyde) for 30 min in the same method as described for the fluorescence confocal microscopy and permeabilized for 20 min. Subsequently, 6.5% bovine serum albumin was added to block the cells for 40 min. Anti-LAMP2/green fluorescent protein-LC3 (GFP-LC3)/p62 antibodies were added for incubation, and then FITC/tetramethylrhodamine-conjugated secondary antibodies were used for fluorescent staining. Cells were stained with DAPI to visualize the nuclei, where the green dots indicate LAMP2 staining, whereas the red dots indicate GFP-LC3/p62 staining. The observed yellow dots, due to the merger of the red and green channels, represent autophagosomes. Finally, immunofluorescence was analyzed under a confocal microscope (Leica TCS SPE, Leica, Wetzlar, Germany). In this experiment, the final concentrations of shRNA1 and HAuCl_4_ were 3 ng/µL and 5μM, respectively, and the total volume in the laser confocal culture dish was 2 mL.

### 2.13. Orthotopic Tumor Model

We purchased several four-week-old BALB/c athymic nude mice from SPF (Beijing) Biotechnology Co. Ltd., Beijing, China and established the tumor model to simulate the natural cancer microenvironment. All animals were kept strictly by the standards and followed the guidance of the Southeast University Animal Research Ethics Committee to conduct all experiments involving mice. HepG2 cells (5 × 10^7^) in 100 µL PBS were injected into the left side of the mouse abdomen using a sterile syringe (1 mL).

When the tumor reached a diameter of about 3 mm, 16 tumor-bearing mice were randomly subdivided further into four groups, and four different preparations were injected five times intravenously every three days. Normal saline (control, 100 µL), free shRNA1 (40 μg), Au NCs (2 mM HAuCl_4_, 100 μL), and Au–shRNA1 NCs (2 mM HAuCl_4_, 100 μL; 40 μg shRNA1) were injected into the four groups (*n* = 4 mice per group). Then, in vivo fluorescence imaging was performed at 0, 12, 24, and 48 h, and the wavelength of the excitation filter was 460 nm. The mice were anesthetized with 2% isoflurane gas, observed with an IVIS Lumina XRMS Series III (Perkin Elmer, Waltham, MA, USA), and the experimental results were recorded. In addition, the body weight of the mice and tumor volume needed to be measured and observed every three days. At the end of the treatment cycle (day 15), all mice were euthanized. The tumor xenografts were harvested and strictly weighed and the main organs dissected for further analyses. Finally, we detected the levels of autophagy-related molecules in different groups of the tumor xenografts by western blotting.

### 2.14. Statistical Analysis

In this study, the statistical analysis software used included GraphPad Prism 8.0 and Origin 8.5. We tested the normality and the variance homogeneity of the data, which were shown as mean ± standard deviation (SD). All experiments required three biological replicates. We used the Student’s *t*-test to compare differences between the means of the two groups. Two-way analysis of variance was used to make paired observations and repeat measurements over time. Significance in statistical analysis was defined as *p* < 0.05.

## 3. Results and Discussion

### 3.1. Elevated Expression of MALAT1 Identified in HCC Patients and HCC Cell Lines

In recent years, a cancer-specific data repository called Oncomine [33,34] has been created and has been of enormous utility for cancer researchers. The MALAT1 expression in liver cancer was compared with those in normal samples using the Oncomine online database. The list was obtained using a meta-analysis of Oncomine data (Figure S1a). The scoring of MALAT1 overexpression in liver cancer samples (hepatocellular adenoma and HCC, using a set of three studies containing 160 samples) versus normal controls is shown [35,36]. Lnc2Cancer 3.0 is an updated version of the cancer storage system that includes investigational support for human cancer-associated lncRNAs and related data [37]. Lnc2Cancer 3.0 was used to obtain detailed data on MALAT1 in HCC including box plots, stage plots, and survival plots (Figure S1b–d). The relevant observations for MALAT1 in HCC demonstrated higher expression levels of MALAT1 in HCC than the controls (*p* < 0.01). The MALAT1 expression in HCC tumors and corresponding adjacent non-cancer tissues (ANCTs) were evaluated by qRT-PCR. The increased expression level of MALAT1 was observed in HCC samples compared with ANCTs (Figure S1e). Moreover, we found that MALAT1 expression was significantly higher in HepG2 and SMMC-7721 than in L02 (Figure S1f). Next, we analyzed the relationship between MALAT1 expression level and disease progression and prognosis in HCC patients (Table S1). In brief, the above results suggest that MALAT1 might be a high-risk factor for the occurrence and development of HCC.

### 3.2. In Situ Self-Assembly of Au–shRNA NCs

**Figure 1:**
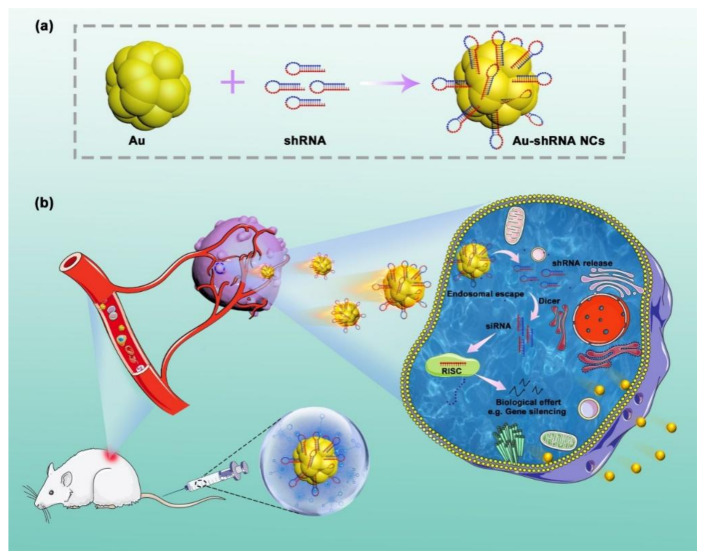
Schematic illustration of the in situ bio-self-assembled fluorescent Au–shRNA NCs to achieve biological effects for cancer imaging and theranostics.

Based on the above observations, we investigated the possibility of utilizing the specific pathological environment of HCC for the in situ bio-self-assembled Au–shRNA NCs to achieve biological effects (e.g., RNAi) for target cancer theranostics. As we know, due to different pH values, tumor cells/tissues will spontaneously produce a large amount of active substances, which causes the tumor microenvironment to be different from normal tissues [38,39]. The unique characteristics of the tumor microenvironment can be exploited by in situ imaging due to the presence of relatively high amounts of specific agents that can act as reducing agents of gold ions for producing fluorescent Au NCs [40,41,42]. Similar to those of siRNA, the related bases of shRNA are negatively charged [43]. Thus, positively charged Au(III) salt reduction can readily attach to negatively charged shRNA, leading to efficient shRNA intracellular transfection to construct fluorescent Au–shRNA NCs. Meanwhile, we observed that in the unique microenvironment of cancer cells, the fluorescent Au–shRNA NCs can readily self-assemble to facilitate tumor bioimaging and treatments, especially when realizing precise RNA silencing effects (Figure 1).

### 3.3. Characterization of Au–shRNA NC Uptake and Escape

**Figure 2:**
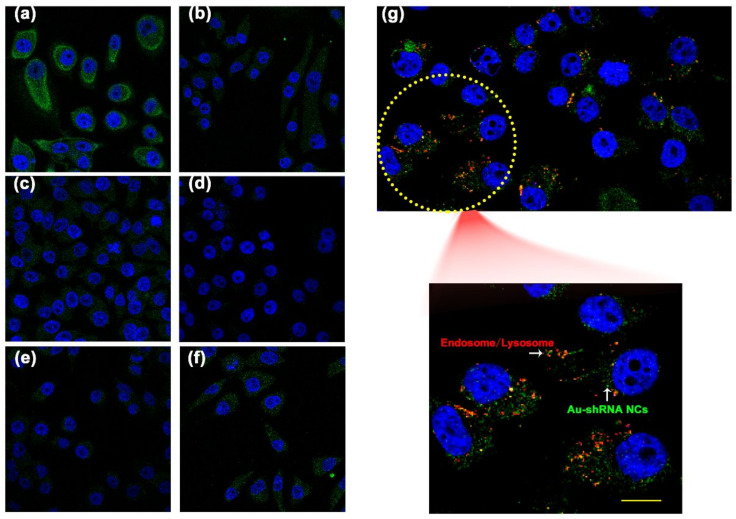
Characterization of Au–shRNA NC uptake and escape. Effect of inhibitors on the uptake of Au–shRNA NCs by live HepG2 cells. When excited at 488 nm, fluorescent confocal images of cells after incubation with Au–shRNA NCs in the absence (**a**) and presence of methyl-β-cyclodextrin (**b**), chloropromazine (**c**), rottlerin (**d**), genistein (**e**), and filipin III (**f**). 4′,6-Diamino-2-phenylindole (DAPI) was used for nucleic staining. (**g**) Colocalization between Au–shRNA1 NCs and endosomes/lysosomes. Green: Au–shRNA1 NCs by 488 nm excitation, Blue: nucleus stained by DAPI; Red: lysosome stained by Lysotracker Red. Scale bar: 10 μm.

To explore possible uptake mechanisms, in this study, we used different inhibitors that inhibit specific endocytic pathways. Methyl-β-cyclodextrin (inhibits lipid-raft-mediated endocytosis), chloropromazine (inhibits clathrin-mediated endocytosis), rottlerin (inhibits macropinocytosis), genistein (inhibits caveolae-mediated endocytosis pathway), and filipin III inhibitors were used in our study. We used laser confocal microscopy to image and excited at 488 nm (Figure 2a–f). The effect of different inhibitors on the uptake of Au–shRNA NC by living HepG2 cells was analyzed in detail, as shown in Figure S2. The results demonstrated that the cells treated with rottlerin significantly reduced the uptake of Au–shRNA NCs compared to the control group. Second, the cells treated with methyl-β-cyclodextrin, chloropromazine, and genistein were also reduced to a certain extent compared with the control. This result suggests that these Au–shRNA NCs are internalized predominantly via the macropinocytosis pathway. Meanwhile, this also suggests a significant role of the lipid-raft-mediated pathway in the uptake, and the caveolae-mediated pathway and clathrin-mediated pathway are also involved in the uptake of Au–shRNA NCs by HepG2 cells.

Endosomal escape is another major factor of the intracellular fate of Au–shRNA NCs after successful cell internalization. Complexes entering the cell via one or more endocytic pathway become entrapped in the vesicles, the vesicles mature, forming early endosomes and late endosomes, and eventually end up in the lysosome. The complexes are effective in achieving endosome escape, otherwise, enzymatic degradation processes take place [44]. Therefore, endosomal escape is also very important for shRNA delivery. If these intracellular nanocomplexes cannot escape from the endosome or lysosome, the Au–shRNA NCs cannot release the encapsulated shRNA into the cytoplasm or nucleus for tumor therapy. Therefore, we further investigated the intracellular distribution and colocalization of Au–shRNA NCs and endosomes/lysosomes by confocal microscopy imaging. Our previous study showed that in situ self-assembly gold nanoclusters in the presence of miRNA/DNA can generate green fluorescence spontaneously at 488 nm [31,32]. Fluorescent cellular images of shRNA and gold salt treated cells (i.e., after co-incubation for 12 h) are shown in Figure 2g. Through imaging, it can be seen that most of the bio-self-assembly Au–shRNA NCs escaped from the endosomes/lysosomes, while the rest were captured, preventing their accumulation in the cytoplasm, which are shown as yellow dots in the image. The position and number of protonable free tertiary amine groups in Au–shRNA NCs may promote the retention of this small part of the nanocomposite [44]. Taken together, with the help of confocal imaging, we intuitively observed that the self-assembled Au–shRNA NC delivery system can successfully realize cellular internalization in targeted cancer cells, and have better endosomal escape capabilities in HepG2 cells.

### 3.4. Effective Silencing of Target Gene MALAT1 via Au–shRNA NCs

**Figure 3:**
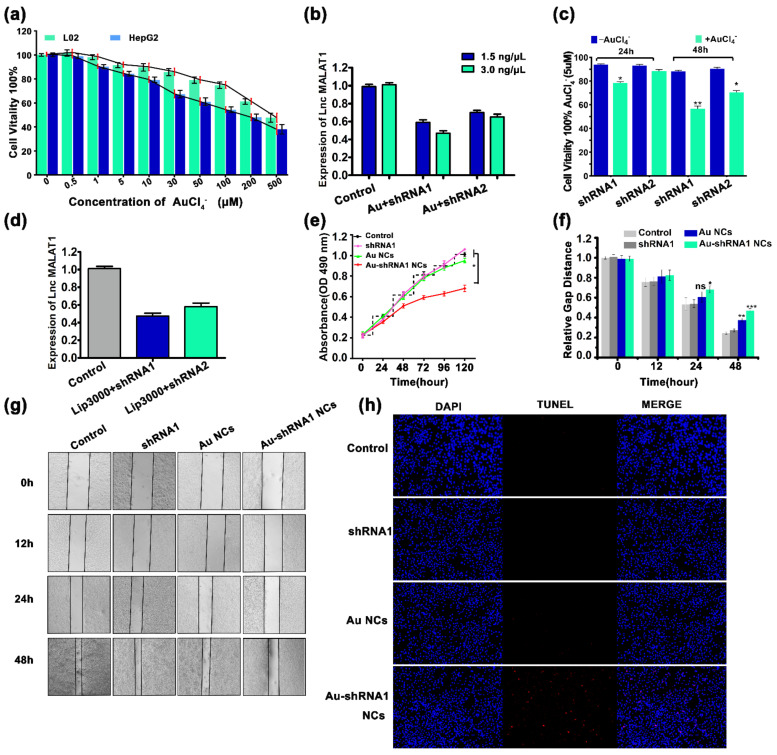
Biochemical characteristics of bio-self-assembled Au–shRNA NCs. (**a**) MTT cell viability and toxicity analysis of HepG2 and L02 cells with HAuCl_4_. (**b**) MTT assay of HepG2 cells with Au–shRNA NCs generated with different concentrations of shRNA1 and shRNA2 (normalized to unprocessed cells). (**c**) The inhibitory effects of shRNA1 and shRNA2 with (green) and without (blue) gold salt in HepG2 cells at 24/48 h treatment. (**d**) The ability of 3 ng/μL shRNA1 and shRNA2 to silence MALAT1 in HepG2 cells was examined by Lip 3000 transfection. (**e**) Long-term (5-day) MTT proliferation analysis of HepG2 cells under different conditions (ANOVA, * *p* < 0.05). Control group (unprocessed), shRNA1 group (shRNA1, 3 ng/μL), Au NCs group (HAuCl_4_, 5 μM), and Au–shRNA1 NC group (shRNA1, 3 ng/μL; HAuCl_4_, 5 μM). (**f**,**g**) The corresponding HepG2 cell scratch-healing experimental analysis and morphological images are also displayed. ** *p* < 0.01, *** *p* < 0.001. (**h**) HepG2 cells treated with shRNA1 with or without gold salt were analyzed by the TUNEL assay. The red color indicates cell apoptosis, and the blue color indicates HepG2 cell nucleus.

To demonstrate the feasibility of the synthesized Au–shRNA NCs to silence MALAT1 in HCCs, we first performed cytotoxicity testing on L02 and HepG2 cells. These results indicate that gold salt (HAuCl_4_ solution) has outstanding biocompatibility with HepG2 and L02 cells. For HepG2 cells, after 48 h of incubation in HAuCl_4_ with a final concentration of ≤5 μM, cell viability remained greater than 80% (Figure 3a). Based on these observations, we further examined the ability of shRNA1 and shRNA2 to silence MALAT1 in HepG2 cells by incubating the cells with gold salt. According to the instructions, we constructed two optimized concentrations of shRNA (1.5 ng/μL, 3.0 ng/μL). The most obviously silencing effect on MALAT1 was at the concentration of shRNA1 (3.0 ng/μL) (Figure 3b). Analysis of the in situ gold nanotransfection shRNA-mediated inhibition indicated that not only was the effect in the presence of HAuCl_4_ notably higher than that without HAuCl_4_, but the effect of shRNA1 with HAuCl_4_ solution was also better than that of shRNA2 with HAuCl_4_. In addition, the inhibitory effect at 24 h was obviously lower than that at 48 h (Figure 3c). Moreover, we examined the ability of shRNA1 and shRNA2 at concentrations of 3.0 ng/μL to silence MALAT1 in HCC cells by the Lipofectamine 3000 Transfection Reagent (Invitrogen, USA, Lip 3000) (Figure 3d). Consistent with the above results, shRNA1 was better than shRNA2 in silencing MALAT1 by Lip 3000. Thus, shRNA1 was selected for MALAT1 silencing in subsequent experiments at a concentration of 3.0 ng/μL.

### 3.5. Cell Proliferation Inhibition and Apoptosis via Au–shRNA1 NCs

The cell proliferation of the Au–shRNA1 NCs group was significantly lower than that of the non-treatment and Au NC group in the 5-day MTT assay (Figure 3e). Through scratch healing experiments, we found that the migration of HepG2 cells with Au–shRNA1 NCs was significantly decreased (Figure 3f,g). Moreover, the percentage of TUNEL positive nuclei (31.16%) in the Au–shRNA1 NC group was significantly higher than the control (0.66%) and the Au NC (4.66%) group (*p* < 0.05; Figure 3h and Figure S3). These observations demonstrate that the generated bio-responsive self-assembling biosynthetic Au–shRNA1 NCs could enhance apoptosis and retard the migration and proliferation of cancer cells.

### 3.6. Physicochemical Characteristics of Bio-Self-Assembled Au–shRNA1 NCs

**Figure 4:**
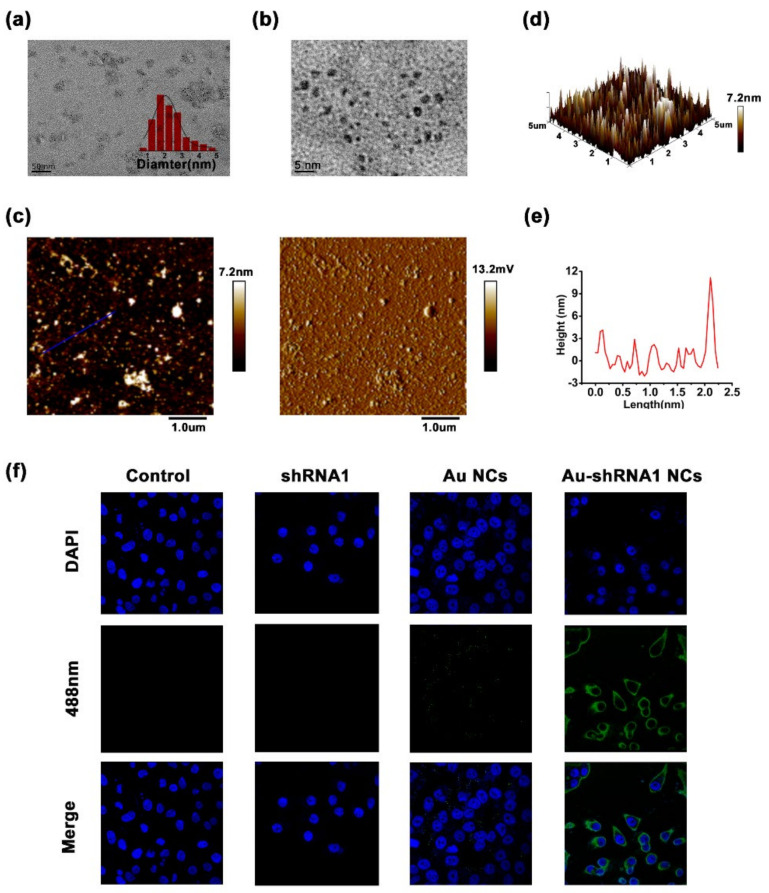
Physicochemical characteristics of bio-self-assembled Au–shRNA1 NCs. (**a**) Typical TEM and (**b**) a higher magnification TEM image of Au–shRNA1 NCs obtained from HepG2 cells after 48 h of culture with HAuCl_4_ (5 μM) and shRNA1 (3 ng/μL). (**c**) Typical AFM height diagram (**left**) and corresponding phase diagram (**right**) of the isolated Au–shRNA1 NCs. (**d**) A 3D model diagram corresponding to (**c**). (**e**) Height analysis of the underlined area in (c) (**left**). (**f**) Laser confocal fluorescence images of HepG2 cells cultured with DMEM, the shRNA1, gold salt, or both the shRNA1 and gold salt. After excitement at 488 nm, visualization of biosynthetic fluorescent Au–shRNA1 NCs in HepG2 cells by fluorescence imaging DAPI was used for nucleic staining. In the above tests, the concentration of shRNA1 was 3 ng/μL, while that of HAuCl_4_ was 5 μM.

To verify the conformation of Au–shRNA1 NCs, we harvested cytoplasmic extracts from cells and further characterized them by TEM and AFM. The TEM image clearly shows the in situ self-assembly Au–shRNA1 NCs in the HepG2 cell extract (Figure 4a,b). When shRNA1 was added, Au NCs with a diameter of about 2–3 nm were clearly visible. This is consistent with our previous research results on gold nanoclusters in the presence of miRNA/DNA [31,32]. Figure 4c–e shows the AFM images of self-assembled biosynthetic Au–shRNA1 NCs isolated from HepG2 cells that had been incubated with shRNA1 and gold salt. The height analysis of the scribe part of the AFM diagram shows that the cumulative height of Au–shRNA1 NCs appears to be approximately 2–3 nm, and the above result is consistent with the TEM characterization. Moreover, HepG2 cells can spontaneously form fluorescent Au NCs under 488 nm excitation by laser confocal fluorescence microscopy. The existence of self-assembled Au–shRNA1 NCs was successfully indicated by green fluorescence inside the cells, and were well dispersed around the nucleoli of the cells, where DAPI were used to stain the nuclei (Figure 4f). We observed that the intracellular fluorescence intensity of the Au–shRNA1 NCs culture group was higher than that of the gold salt-only culture group. This observation suggests that these Au–shRNA1 NCs can enhance intracellular fluorescence. In contrast, such fluorescent characteristics were not observed in L02 cells under all experimental conditions (Figure S4).

### 3.7. Inhibition of Autophagic Flux through Silencing of MALAT1 by Au–shRNA1 NCs

**Figure 5:**
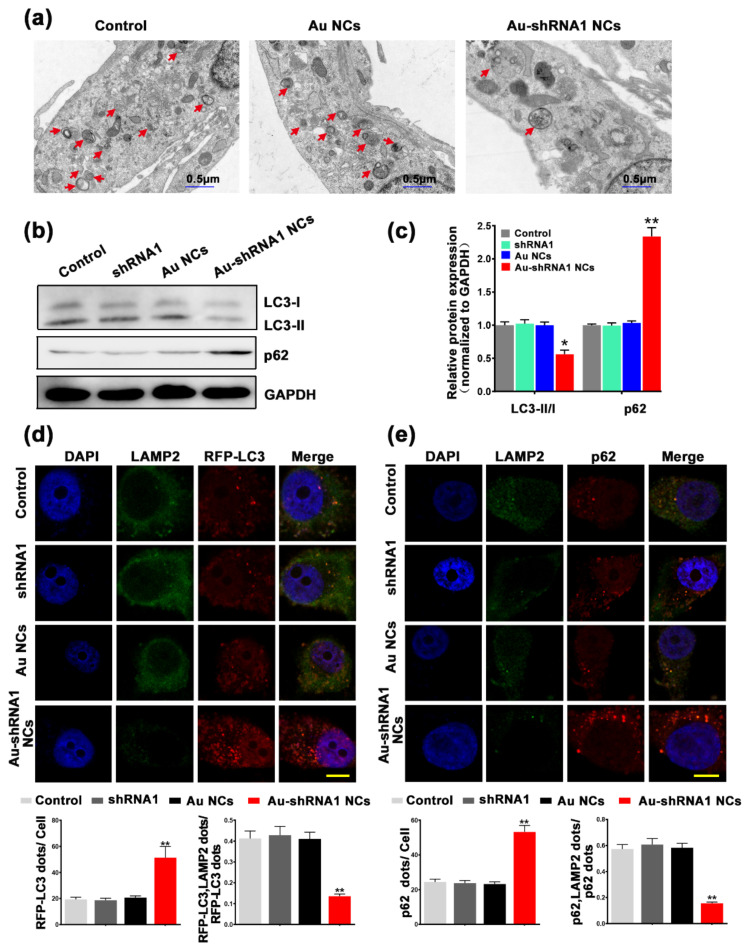
Silencing of MALAT1 by Au–shRNA1 NCs inhibits autophagic flux. (**a**) Bio-TEM images of HepG2 cells showing the accumulation of autophagosomes with or without gold salt treatment (bars = 0.5 μm). Red arrows indicate autophagosomes. (**b**,**c**) Western blot analysis was used to compare the expression levels of LC3 and p62 in the untreated control group, shRNA1, Au NC, and Au–shRNA1 NC groups. * *p* < 0.05, ** *p* < 0.01 (*n* = 3–5). (**d**) Representative images show RFP-LC3 and LAMP2 expression among different groups of HepG2 cells. Cells were first transfected with RFP-LC3 for 24 h, and then separately incubated with the shRNA1, gold salt, or the shRNA1 and gold salt together for 12 h. DAPI staining was applied to observe the cell nucleus. Scale bar: 5 μm. The figure shows the quantification of RFP-LC3 (red) dots. The bottom graph shows the combined point/RFP-LC3 ratio. ** *p* < 0.01 (*n* = 3–5). (**e**) Representative images of p62 and LAMP2 between different groups. The yellow dots in the merged image indicate the co-localization of LAMP2 and p62. Scale bar: 5 μm. The quantitative analysis of p62 (red) spots/cells and fusion spots/p62 in HepG2 cells is indicated (bottom). ** *p* < 0.01 (*n* = 3–5).

Studies suggest that autophagy is associated with poor clinical prognosis of certain cancers, and inhibition of autophagy has been shown to reduce cancer growth [11,28,45]. Biological imaging is an important research method to understand the tissue structure of organisms and clarify various physiological functions of organisms. In the current study, the bio-TEM image was performed to confirm the formation of autophagosomes in different groups. The bio-TEM image shows that the whole cell was a long spindle shape, the edge of the cell membrane was relatively complete, the cell matrix was evenly distributed, and the organelles were abundant. The noticeable difference was that there were fewer autophagosomes in the Au–shRNA NC group compared to the other groups (red arrow). Our findings revealed an obvious decrease in the cytoplasmic structures of autophagosomes and autolysosomes and lysosomes in HepG2 cells after co-incubation with gold salt and the shRNA1 (Figure 5a). Compared with the control group and the Au NC group, the autophagy level of the Au–shRNA1 NC group was reduced. Western blot image analysis also supports this result (Figure 5b,c). In order to explore the correlation between MALAT1 silencing and autophagy flux in HepG2 cells, western blot analysis was performed to detect LC3 to determine the abundance of autophagosomes in the cytoplasm. LC3 is currently recognized as a marker for autophagy [46]. During the formation of autophagy, the cytoplasmic LC3 (i.e., LC3-I) will enzymically decompose a small segment of the membrane and transform it into (autophagosome) membrane (i.e., LC3-II). The ratio of LC3-II/I can estimate the level of autophagy. p62 can connect LC3 and ubiquitinated substrates, and then be integrated into autophagosomes, and degraded in autophagolysosomes, so it is used as an indicator of autophagy degradation [47]. Therefore, it can be considered that the decrease in the ratio of LC3-II/I and the increase in p62 level during the autophagic flux of organisms indicate autophagy inhibition. LAMP2 is a lysosomal membrane protein that can be used to monitor autophagosome and lysosome fusion. Our results showed that compared with the control group and the Au NC group, the Au–shRNA1 NC group had a significantly lower LC3-II/I level, while the p62 level was significantly increased.

Interestingly, confocal immunofluorescence imaging of a single tumor cell, as shown in Figure 5d, co-incubating gold salt and the shRNA1 similarly increased the number of RFP-LC3 positive dots (red), while decreased the number of LAMP2 positive spots (green). The number of p62 positive spots increased in shRNA1 and gold salt transfected cells, while the ratio of p62-LAMP2 pooled (merge) spots/p62 spots decreased compared to other cells (seen in Figure 5e). In addition, RFP-positive/p62 puncta were partly colocalized with LAMP2 in HepG2 cells co-incubated with gold salt and the shRNA1. Through bio-TEM and confocal imaging, the dynamics of autophagy flux and the expression changes of autophagy molecular markers in tumor cells are tracked to realize complex dynamic spatiotemporal analysis. Together, these results demonstrate that silencing of MALAT1 by Au–shRNA1 NCs inhibits autophagic flux.

### 3.8. Au–shRNA1 NCs for Effective Bioimaging and Theranostics in an Orthotopic Tumor Model

**Figure 6:**
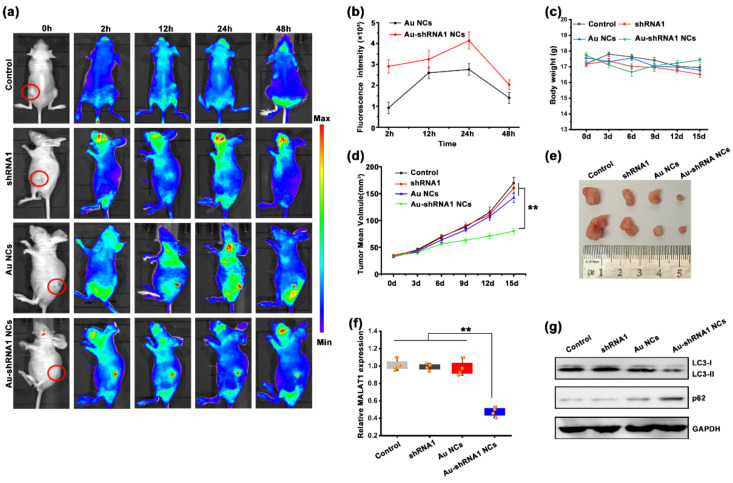
Silencing of MALAT1 by Au–shRNA1 NCs inhibits tumor proliferation in orthotopic tumor model via suppression of autophagic flux. (**a**) Dynamic biodistribution of normal saline (control), shRNA1, Au NCs, and Au–shRNA1 NCs in mice using fluorescent imaging at 0, 12, 24 and 48 h. (**b**) Fluorescence imaging of nude mice bearing HepG2 tumors at various time points after the injection of HAuCl_4_ (black) or HAuCl_4_ and shRNA1 (red) (*n* = 4). (**c**) Weights of each group of mice during the 15-day treatment. (**d**) HepG2 tumor growth in different time courses after treatment (ANOVA, ** *p* < 0.01). (**e**) Tumor images on day 15. (**f**) The expression of MALAT1 in tumor tissues of different treatment groups was detected by the qRT-PCR assay (** *p* < 0.01). (**g**) The expression of autophagy markers (LC3 and p62) in tumor tissues of different treatment groups was detected by western blot assay.

Based on the cell experiments, we speculated that MALAT1-silenced cells had considerably lower pro-tumorigenic functions through reduced autophagy. To better understand and simulate the therapeutic effect of the synthesized Au–shRNA1 NCs, a xenograft tumor model was developed for further assessment. We inoculated xenograft tumors by injecting HepG2 cells and successfully established HCC tumor models (Figure S5a). We randomly divided the tumor model mice into four groups (i.e., the control, Au NC, shRNA1, and biosynthesized Au–shRNA1 NC groups), with four mice per group. We first explored whether self-assembled Au–shRNA1 NCs could be effectively delivered to tumors using real-time fluorescence imaging. According to the different experimental designs, the groups of mice were injected with different substances, and images were collected at 2–48 h (Figure 6a). The fluorescence intensity in the tumor tissue at different periods is shown in Figure 6b. The results show that self-assembled Au–shRNA1 NCs can achieve non-invasive fluorescence imaging of live animals and real-time detection of targeted tumors. The average intensity of the fluorescent signal increased with time and reaches a maximum in 24 h. In addition, the fluorescence signal of the Au–shRNA1 NC group was much higher than the Au NC group. Moreover, the resulting fluorescence was more intense in tumors, which further showed that the biosynthesized fluorescent Au–shRNA1 NCs were present in target tumors.

In addition to the above observations, we recorded the weights of the mice (Figure 6c). No remarkable differences were observed between the different groups during the treatment, suggesting few side effects. After five cycles of injection treatment, all nude mice were euthanized and xenograft tumors were collected for further analysis. Consistent with the results obtained in the in vitro cell experiment, the self-assembly biosynthesis Au–shRNA1 NC group showed enhanced inhibition of tumor growth (Figure 6d). The in situ self-assembled Au–shRNA1 NC treated group significantly reduced tumor volume (Figure 6e and Figure S5b,c). In addition, ex vivo imaging of mice treated with in situ synthesized Au NCs and Au–shRNA1 NCs showed that gold was mainly eliminated in vivo by the liver and kidneys (Figure S5d), which is consistent with previous reports [48,49].

Furthermore, we tested the mRNA levels of MALAT1 in different groups of tumor tissues to verify the ability of Au–shRNA1 NCs to silence MALAT1. As shown in Figure 6f, compared with that in the control groups treated with PBS, shRNA1 alone, or gold salt alone, the mRNA level of MALAT1 in the Au–shRNA1 NC group was significantly reduced. We then focused on the expression levels of p62 and LC3-II/I, which were measured in the aforementioned studies. The depletion of MALAT1 inhibited autophagy in tumor cells, and the western blot results showed that the LC3-II/I level in the Au–shRNA1 NC group was significantly reduced, while the p62 level was significantly increased compared with those in the control groups (Figure 6g). Moreover, to further evaluate the safety of the complex in vivo, we tested the biochemical parameters in the blood after the mice were killed (Figure S5e–i) and performed hematoxylin-eosin staining of the major organs (Figure S6). The results showed that the in situ biosynthetic Au–shRNA1 NCs had no obvious toxicity and did not cause damage to the liver or kidneys in mice.

## 4. Conclusions

shRNAs/siRNAs have great promise in disease treatment as potential drugs for silencing disease-related genes. However, due to the lack of effective and safe carriers, their usage is restricted. For cancer treatment, many major breakthroughs have been made in the past two decades, but there are still huge challenges. There is an urgent need to introduce safe and effective approaches for real-time bioimaging and monitoring of tumor development and treatments. At present, the application of bioimaging technology in clinical medical diagnosis has attracted much attention. The development of non-invasive in vivo imaging technology is an important prerequisite for its wide application in disease diagnosis and treatment. Furthermore, the participation of new fluorescent materials such as bio-self-assembly nanomaterials makes the application of imaging technology more accurate and biocompatible. Fluorescent nanomaterials have received increasing attention due to their unique physicochemical properties, and were used in medicine and other fields [50]. Nanocarriers as drug delivery systems are promising and have increased in popularity, especially for cancer treatment. Thus, with the help of bio-responsive molecular-level bioimaging technology, the development of a non-toxic, safe, and effective gold nanoparticle delivery system for shRNA/siRNA is critical to the clinical success of gene therapy.

Herein, we propose a novel method for shRNA delivery, imaging, and treatment of cancers using bio-responsive self-assembled fluorescent Au–shRNA NCs. It has significant advantages such as high targeting efficiency and high biocompatibility in precise tumor bioimaging and drug delivery systems. Its advantages in regulating cytotoxicity, cellular uptake, endosomal escape, and shRNA transfection efficiency may come from changing the balance between modules with different functions (e.g., electrostatic charge and pH) [51,52]. In situ self-assembled Au–shRNA NCs can protect shRNA from external effects, realize cellular uptake, and effective endosomal escape. In addition, the TEM and AFM images as well as the fluorescent characterization of these complexes, provide consistent evidence of in situ self-assembling Au–shRNA1 NCs. These observations support the formation of bio-responsive Au–shRNA1 NCs in vivo that form specifically in the unique cancer microenvironment [42,53,54].

**Figure 7:**
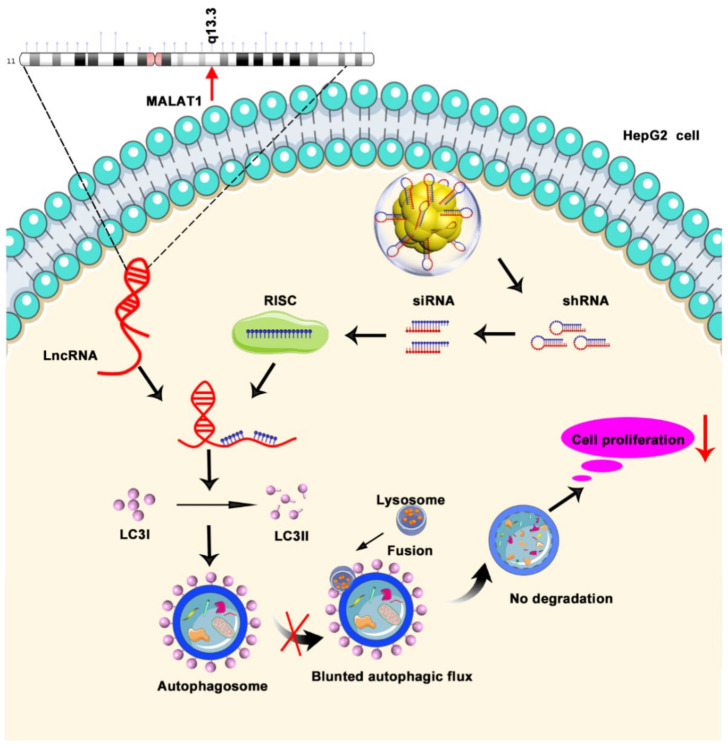
Schematic diagram of the potential mechanism by which Au–shRNA1 NCs effectively silence MALAT1 to inhibit the proliferation of HepG2 cells via the suppression of autophagy.

Studies have demonstrated that MALAT1 is involved in the autophagy pathway and may be an inducer of autophagy [28,55,56]. More evidence shows that autophagy can help cancer cells overcome stress conditions, and tumor cells rely on autophagy as a survival strategy [57]. In this study, we used bio-self-assembled Au–shRNA1 NCs to silence MALAT1 and observed the resulting biological effects. In situ Au–shRNA1 NCs self-assembled in HepG2 HCC cells, and their various conformation states were further proven by TEM and AFM characterization. Furthermore, from a biological point of view, through a series of in vivo and in vitro related experiments and biological imaging, our observations demonstrated that the self-assembled fluorescence Au–shRNA1 NCs effectively bioimaged the diseased locations and silenced MALAT1, inhibiting the proliferation of HepG2 cells by suppressing autophagic flux (Figure 7).

In summary, based on the observations above, our results demonstrate that the bio-responsive self-assembly Au–shRNA NCs could readily realize real-time cancer cell imaging and precise monitoring of tumor-targeting treatment from multi-scale levels, which can be further used to guide targeted cancer therapy. To the best of our knowledge, it is the first example to report a new shRNA self-assembled for a targeted nano-delivery system from the genetic level for non-invasive and effective cancer bioimaging and treatment. MALAT1 is one of the star molecules of lncRNA and is upregulated in HCC; in this study, MALAT1-shRNA was first utilized to exploit the considerable efficiency of the in-situ bio-responsive self-assembly Au–shRNA NCs on silencing target gene MALAT1, which led to significant changes in autophagy. Meanwhile, when combined with bio-TEM and laser confocal imaging studies to track the dynamic changes of autophagic flux caused by the as-prepared Au–shRNA1NCs, it is exciting to realize high-resolution complex dynamic spatiotemporal analysis readily. This raises the possibility of facilitating accurate multi-scale bio-imaging from the molecular-level with target gene-recognition to cancer cell imaging and in vivo tumor tissue imaging for simultaneous precise targeted cancer therapy. In the future, we can further increase the sample size and more cell lines, combining them with the corresponding clinic samples. Thus, we believe that the ongoing cutting-edge studies will eventually provide a unique and promising theranostics strategy for cancer early diagnosis and precision treatment.

## Data Availability

Not applicable.

## Supplementary Materials

The following are available online at https://www.mdpi.com/article/10.3390/bios11110425/s1, Figure S1: Elevated expression of MALAT1 identified in HCC. Figure S2: Analysis of the effect of different inhibitors on the uptake of Au–shRNA NCs by live HepG2 cells. Figure S3: Analysis of the tumor cell apoptosis rate in different groups (TUNEL staining). Figure S4: Representative laser confocal fluorescence images of L02 cells cultured with shRNA1 and gold salt. Figure S5: Au–shRNA NCs to achieve biological effects for tumor mice imaging and theranostics. Figure S6: Tissue sections of major dissected organs from different treatment groups were stained with H&E. Table S1: Correlations between MALAT1 expression and clinicopathological characteristics in 30 HCC tissues. Table S2: Sequences of shRNA and lncRNA primers. Table S3: The antibodies employed for western blot analysis.
