# Supplementary material for: Intelligent Bio-Responsive Fluorescent Au–shRNA Complexes for Regulated Autophagy and Effective Cancer Bioimaging and Therapeutics
Source: Biosensors (Basel). 2021 Oct 28;11(11):425. doi: 10.3390/bios11110425 (PMC8615530; doi:10.3390/bios11110425)
Supplement: Supplementary file 1 [file biosensors-11-00425-s001.zip › biosensors-1343934-supplementary.pdf]

biosensors

MDPI

Supplementary Materials

Intelligent Bio-Responsive Fluorescent Au–shRNA Complexes for Regulated Autophagy and Effective Cancer Bioimaging and Therapeutics

Weijuan Cai [1], Liang Yin [2], Hui Jiang [1], Yossi Weizmann [3,*] and Xuemei Wang [1,*]

[1] State Key Laboratory of Bioelectronics (Chien-Shiung Wu Lab), School of Biological Science and Medical Engineering, Southeast University, Nanjing 210096, China; 230188127@seu.edu.cn (W.C.); sungi@seu.edu.cn (H.J.)

[2] Department of Endocrinology and Metabolism, Shunde Hospital of Southern Medical University, Shunde 528300, China; yinliang151@sina.com

[3] Department of Chemistry, Ben-Gurion University of the Negev, 8410501 Beer-Sheva, Israel

* Correspondence: Correspondence: yweizmann@bgu.ac.il (Y.W.); xuewang@seu.edu.cn (X.W.)

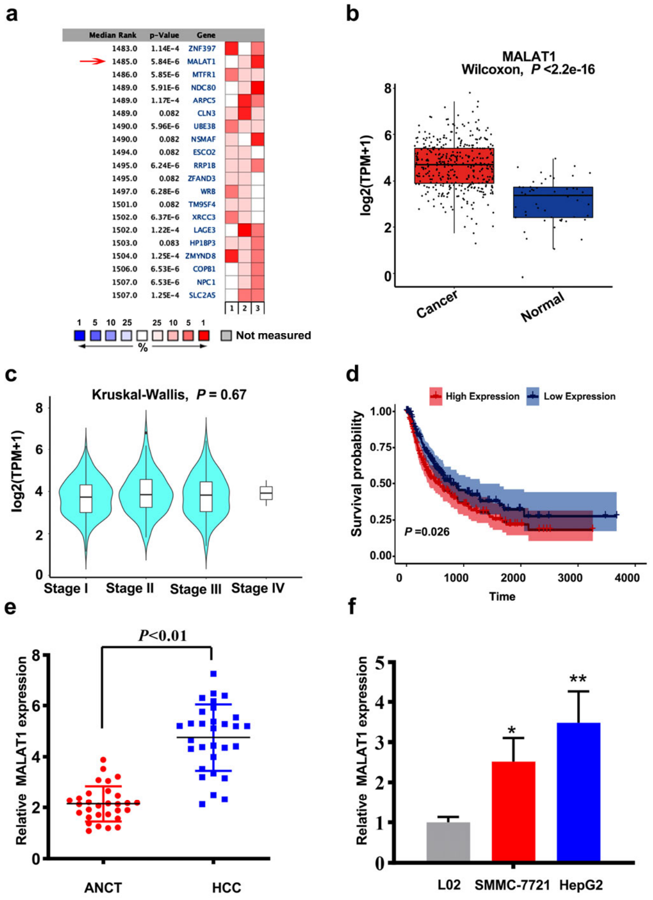

Figure S1. Elevated expression of MALAT1 identified in HCC. (**a**) Upregulated genes were obtained by using the Oncomine database (http://www.oncomine.org) with the following filters: differential analysis, cancer versus normal analysis, and cancer type (liver cancer). The *P*-value for a gene is the *P*-value from the median-ranked analysis. Data for comparison are as shown in the legend. The columns correspond to the following studies: 1,[1] 2,[1] and 3.[2] (**b**–**d**) RNA-seq web tools in Lnc2Cancer 3.0 (http://www.bio-bigdata.net/lnc2cancer)

were used to obtain detailed data on the relationship between HCC and lncRNA-MALAT1, including box plots, stage plots, and survival plot. (**e**) qRT-PCR was used to measure MALAT1 expression and compare HCC tissues (n = 30) and the corresponding adjacent noncancer tissues (ANCTs). (**f**) MALAT1 expression in HepG2/SMMC-7721 cell lines and in normal embryonic hepatocytes (L02). The results are expressed as the mean±SD. *$P < 0.05$, **$P < 0.01$, in comparison with the indicated group.

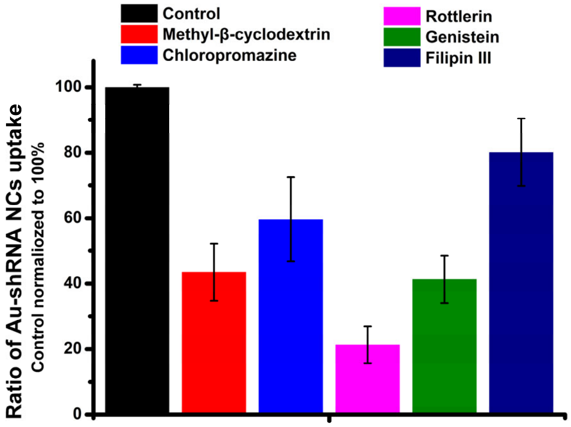

Figure S2. Analysis of effect of different inhibitors on the uptake of Au-shRNA NCs by live HepG2 cells.

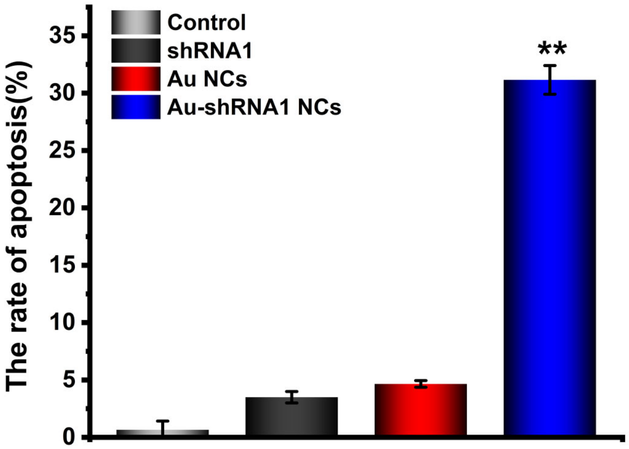

Figure S3. Analysis of the tumour cell apoptosis rate in different groups (TUNEL staining). **$P < 0.01$.

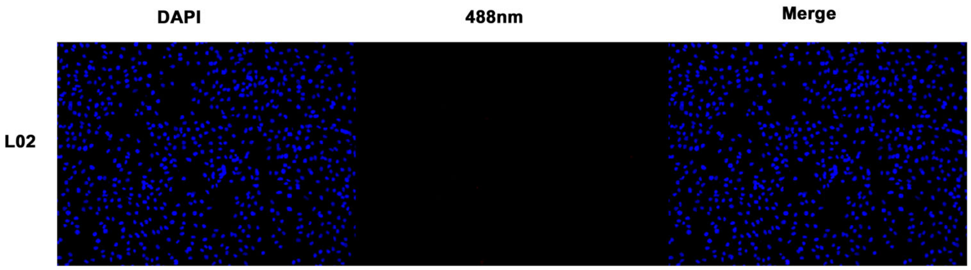

Figure S4. Representative laser confocal fluorescence images of L02 cells cultured with shRNA1 and gold salt. When excited at 488 nm, intracellular fluorescence was not observed under the same experimental conditions. DAPI were used for nucleic staining. Confocal laser scanning microscopy was used for cell visualization.

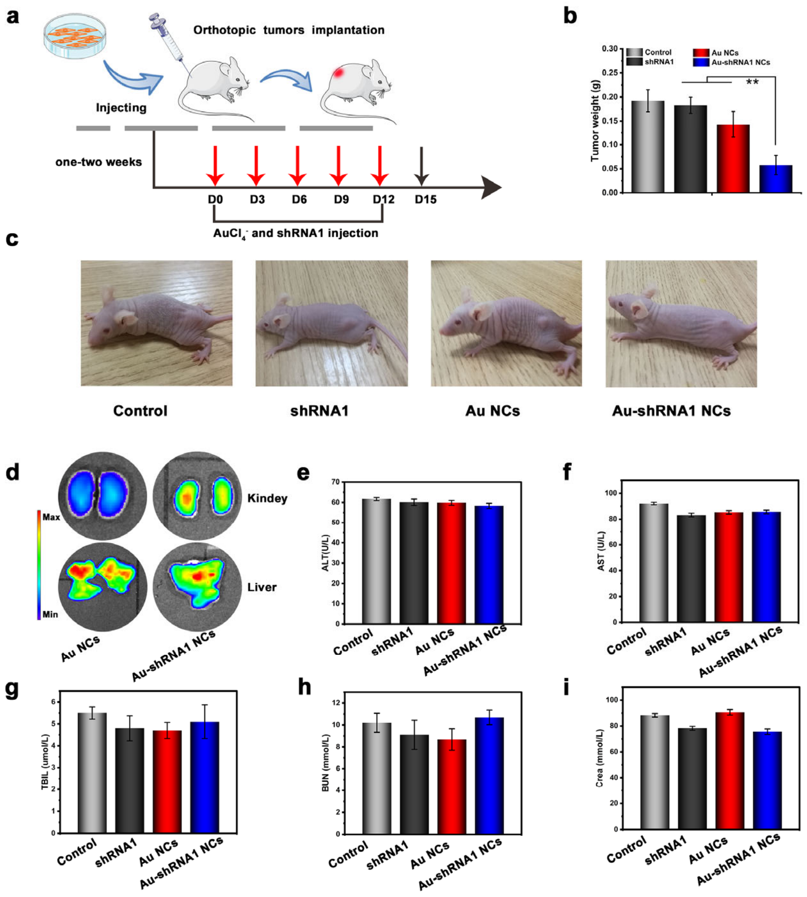

Figure S5. (**a**) Schematic diagram illustrating the primary nude tumour mouse model and design of the experimental treatment of tumour mice. (**b**) Weights of isolated tumors in each group. (**c**) Images of mice after 15 days of different treatments. (**d**) Representative images of dissected organs (liver and kidneys) from HepG2 tumour-bearing mice from the Au NCs group and Au-shRNA1 NCs group. (**e**–**i**) After the mice were euthanized, the results of biochemical indicators of liver and kidney function in the blood of different groups were obtained.

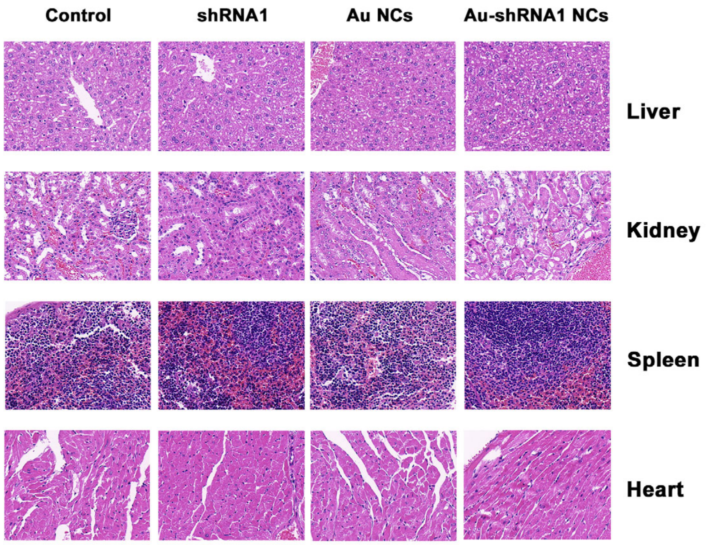

Figure S6. Tissue sections of major dissected organs from different treatment groups were stained with H&E (20×).

Table S1. Correlations between MALAT1 expression and clinicopathological characteristics in 30 HCC tissues.

Characteristic	n	MALAT1 expression		*P* value
		Low	High	
**All case**	**30**	7	23	
**Gender**				
Male	22	4(18.2%)	18(81.8%)	0.345
Female	8	3(37.5%)	5(62.5%)	
**Age**				1.000
≥60	24	6(25.0%)	18(75.0%)	
<60	6	1(16.7%)	5(83.3%)	
**liver disease**				
HBV	23	5(21.7%)	18(78.3%)	1.000
HBV+ others	7	2 (25.0%)	5(75.0%)	
**Tumor number**				
Single	13	4(30.8%)	9(69.2%)	0.666
Multiple	17	3(17.6%)	14(82.4%)	
**Tumor size (cm)**				
>5	19	1(5.3%)	18(94.7%)	0.032*
≤5	11	6(37.5%)	10(62.5%)	
**TNM stage**				
I + II	17	2(11.8%)	15(88.2%)	0.190
III + IV	13	5(38.5%)	8(61.5%)	
**Venous invasion**				
with	17	1(5.9%)	16(94.1%)	0.025*
without	13	6 (46.2%)	7(53.8%)	
**Distant metastasis**				
with	19	1(5.3%)	18(94.7%)	0.004**
without	11	6(54.5%)	5(45.5%)	
**BCLC stage**				
A	14	5(35.7%)	9(64.3%)	0.204
B+C+D	16	2(12.5%)	14(87.5%)	
**AFP levles (U/L)**				
>20	20	6(25.0%)	14(75.0%)	0.372
≤20	10	1(16.7%)	9(83.3%)	

*P<0.05, **P<0.01; HBV: hepatitis B virus; TNM: tumour, node and metastasis; BCLC: Barcelona Clinic Liver Cancer; AFP: alpha-fetoprotein.

Table S2. Sequences of shRNA and lncRNA primers.

		Sequences 5′ → 3′
Primers qRT-PCR MALAT1	F primer	AAAGCAAGGTCTCCCCACAAG
	R primer	GGTCTGTGCTAGATCAAAAGGC
GAPDH	F primer	TCTCTGCTCCTCCTGTTCGA
	R primer	GCGCCCAATACGACCAAATC
MALAT1 shRNA1		GGCAGCTGTTAACAGATAAGT
shRNA2		GCCGAAATAAATGAGAGATGA
shRNA control		GGGTGAACTCACGTCAGAA

Table S3. The antibodies employed for the western blot.

Antibody	Working dilutions
LC3	1:1000
	1:100
p62	1:1000
	1:100
GAPDH	1:1000

References

1. Chen, X.; Cheung, S.T.; So, S.; Fan, S.T.; Barry, C.; Higgins, J.; Lai, K.M.; Ji, J.; Dudoit, S.; Ng, I.O.; et al., Gene expression patterns in human liver cancers. *Mol. Biol. Cell.* **2002**, *13*, 1929–1939.
2. Wurmbach, E.; Chen, Y.B.; Khitrov, G.; Zhang, W.; Roayaie, S.; Schwartz, M.; Fiel, I.; Thung, S.; Mazzaferro, V.; Bruix, J.; et al., Genome-wide molecular profiles of HCV-induced dysplasia and hepatocellular carcinoma. *Hepatology.* **2007**, *45*, 938–947.
